# Antecubital Fossa Solitary Osteochondroma with Associated Bicipitoradial Bursitis

**DOI:** 10.1155/2015/560372

**Published:** 2015-08-27

**Authors:** Colin Ng, Luigi Bibiano, Stephan Grech, Branko Magazinovic

**Affiliations:** ^1^Department of Trauma and Orthopaedics, Mater Dei Hospital, Triq Dun Karm, Msida MSD 2090, Malta; ^2^Clinica Ortopedica, Seconda Università degli Studi di Napoli, 4 Via De Crecchio, 80138 Napoli, Italy

## Abstract

Antecubital fossa lesions are uncommon conditions that present to the orthopaedic clinic. Furthermore, the radius bone is an uncommonly reported location for an osteochondroma, especially when presenting with a concurrent reactive bicipitoradial bursitis. Osteochondromas are a type of developmental lesion rather than a true neoplasm. They constitute up to 15% of all bone tumours and up to 50% of benign bone tumours. They may occur as solitary or multiple lesions. Multiple lesions are usually associated with a syndrome known as hereditary multiple exostoses (HME). Malignant transformation is known to occur but is rare. Bicipitoradial bursitis is a condition which can occur as primary or secondary (reactive) pathology. In our case, the radius bone osteochondroma caused reactive bicipitoradial bursitis. The differential diagnosis of such antecubital fossa masses is vast but may be narrowed down through a targeted history, stepwise radiological investigations, and histological confirmation. Our aim is to ensure that orthopaedic clinicians keep a wide differential in mind when dealing with antecubital fossa mass lesions.

## 1. Introduction

Antecubital fossa mass lesions may be either benign or malignant in nature [1, 2]. Benign conditions include synovial osteochondromatosis [1], brachial artery aneurysms, ganglions, bursitis, and hemangiomas. Malignant tumors may include synovial and muscle sarcomas [2, 3], as well as chondrosarcomas [4, 5].

Osteochondromas are a type of developmental lesion rather than a true neoplasm [6]. They are the most frequent benign bone tumour that occur in metaphyseal regions of long bones [6]. The pathology consists of an atypical growth of cartilaginous tissue of the physis [4, 5, 7]. They are composed of cortical and medullary bone with overlying hyaline cartilage cap which also exhibits continuity with underlying bone cortex and medulla. Murphey et al. [6] state that the continuity of this lesion is pathognomonic.

Osteochondromas are known to exist in the forms of solitary and hereditary multiple osteochondromatosis [6, 8]. Solitary types tend to be asymptomatic and diagnosed incidentally. Symptomatic lesions normally occur in young patients, with up to 80% diagnosed prior to the age of 20, commonly found in the femur, tibia, humerus, pelvis [7, 9], and rarely the elbow. Clinically, they may present with pain, swelling, restricted range of motion, neuropathy, vascular compromise, and abnormal cosmesis [5]. In contrast, HME is inherited in an autosomal dominant pattern and usually occurs and is diagnosed in patients under the age of 5, affecting virtually any bone of the body [6].

Complications of osteochondromas are vast. Frequently occurring examples include mechanical range of motion blocks, nerve impingement, tendon rupture, fracture and deformity, bursitis, extensive growth without malignant change, and malignant transformation of the cartilaginous cap [4, 6–8]. Solitary osteochondromas have a 1-2% risk of developing chondrosarcoma [4]. Secondary chondrosarcoma rarely occurs prior to age of twenty years [5]. On histology, a cartilaginous cap of >2 cm and/or irregularity of the cap in adults generally corresponds to malignant transformation [10, 11].

Bicipitoradial bursitis is a form of chronic bursitis with only a handful of cases documented in the current literature [12–15]. It can affect diverse groups of patients either as a consequence of overuse mechanical injuries [15] or secondary to known pathological processes. It is known to be associated with biceps tendon tear and tendinopathy [16]. Other causes include tuberculosis [17], chemical synovitis, synovial cyst of the anterior elbow capsule [14], psoriatic arthropathy, and rheumatoid arthritis [15]. Elbow movements promote inflammation, swelling, and pressure increases within the bursa [18] which manifests in pain and associated symptoms depending on the bursa's relationship to other anatomical structures.

## 2. Case Report

61-year-old lady, right hand dominant homemaker, presented with a four-year history of right anterior elbow pains localised in the antecubital fossa associated with swelling and intermittent paraesthesia in the radial border of forearm extending towards the thumb limiting her thumb flexion at the distal interphalangeal joint. She denied any forms of trauma and symptoms of osteoarthritis and rheumatoid arthritis and was otherwise systemically healthy. She complained of pain on active elbow flexion and both pronation and supination. Prior to presentation to the orthopaedic outpatient clinic, she described a lengthy period of conservative management through simple analgesia including oral paracetamol, oral nonsteroidal anti-inflammatories, and physiotherapy.

On examination, a palpable mass was felt in the right antecubital fossa measuring roughly 5 cm in diameter fixed to the underlying soft tissue structures. There was pain on active elbow flexion and pronation, with reduction of range of motion in both ranges. Elbow extension and supination were neither painful nor limited.

Strength was equal to the contralateral side; however pain was elicited on all resisted movements. There was no vascular compromise, but reduced cutaneous sensation on the radial side of the thumb, corresponding to the distribution of the superficial radial nerve.

**Figure 1:**
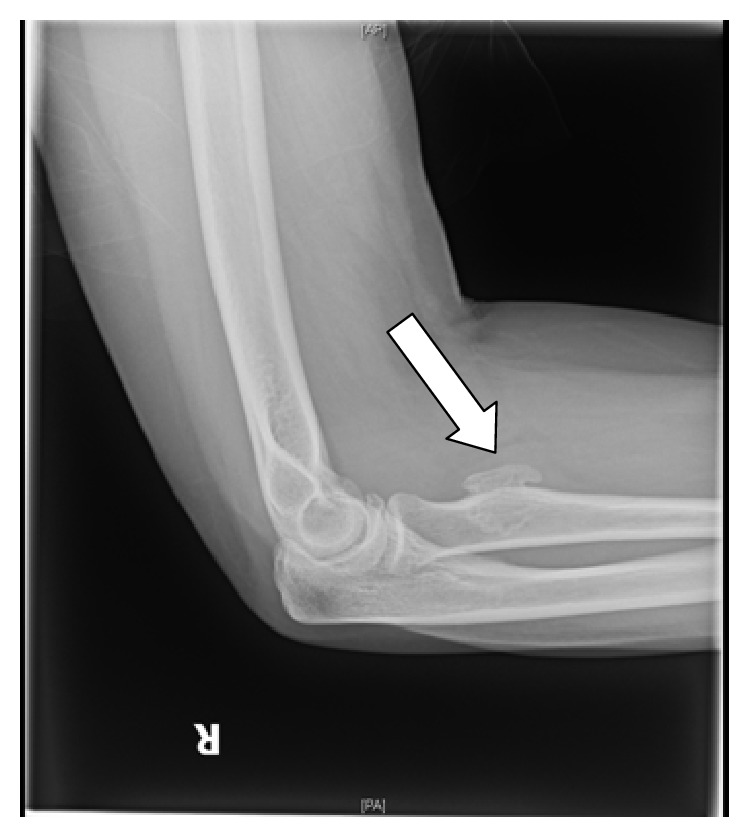
Plain radiograph reported this calcified area as an osteochondroma right forearm (fat arrow).

A plain radiograph was performed (Figure 1) which showed an irregular circumscribed radioopaque lesion overlying the proximal radius, in close proximity to the radial tuberosity and distal to the radial head. It was reported as a possible osteochondroma. In view of this inconclusive diagnosis a computer tomography (CT) scan was then performed. It also reported an indeterminate sessile osteochondroma. However, despite the overall morphology, given that the corresponding medullary cavity appears to be arising off the cortex, a differential diagnosis of a bony enthesopathic reaction was considered; thus magnetic resonance imaging (MRI) was therefore recommended.

**Figure 2:**
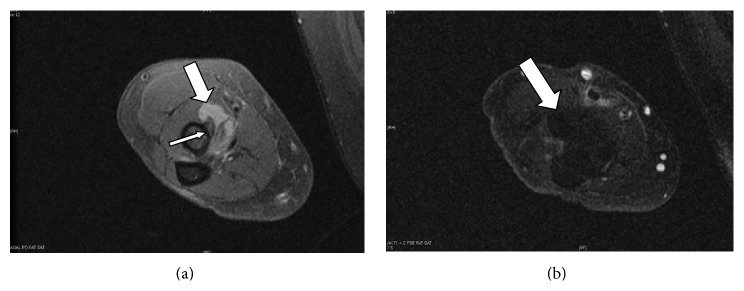
(a) MRI axial T1 weighted before IV gadolinium. Hyperintense signal showing the bursa (Fat arrow) and biceps tendon (thin arrow). (b) MRI axial T1 weighted after IV gadolinium sequences. Bursa (thick arrow).

**Figure 3:**
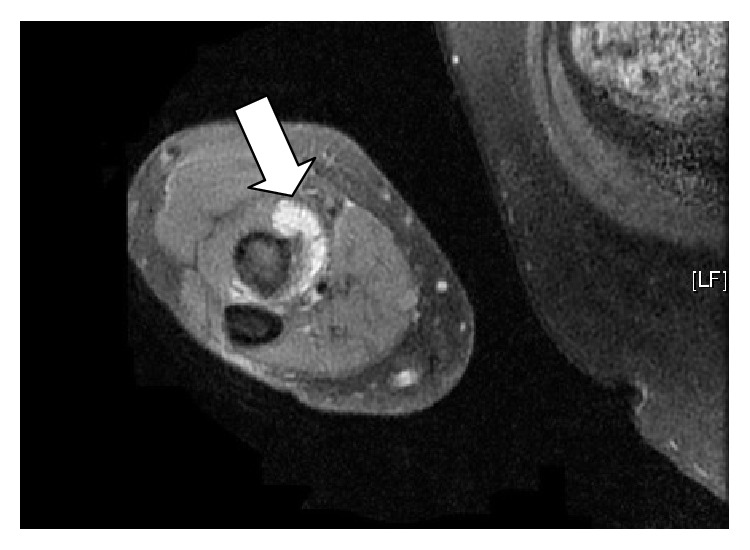
MRI axial T2 weighted image after IV gadolinium clearly highlighting the bursa structure (thick arrow).

MRI before and after gadolinium confirmed the appearance of an 8 mm by 9 mm fluid collection compatible with bicipitoradial bursitis and ongoing synovitis between the biceps brachii tendon and the radial tuberosity (Figures 2(a), 2(b), and 3). Furthermore, there was evidence of distal biceps enthesopathy with associated adjacent bony changes. The biceps tendon was reported to be completely intact.

**Figure 4:**
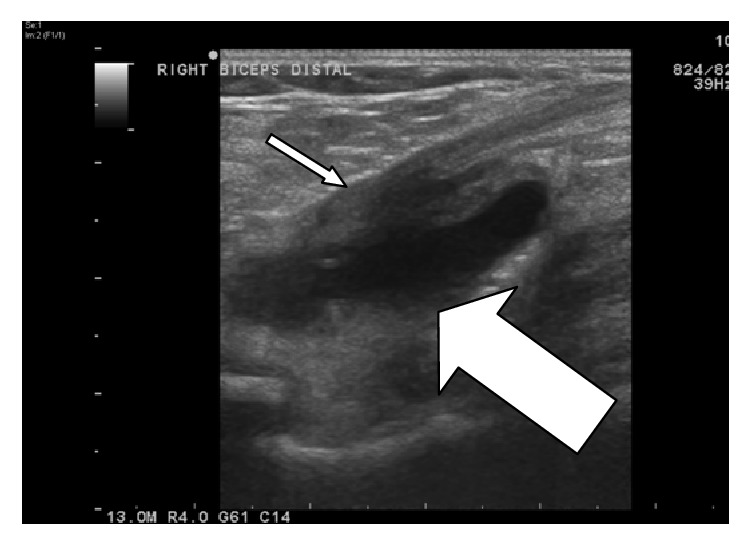
US image. Bicep tendon insertion to radius (thin arrow). Bursa (thick arrow).

An ultrasound guided aspiration was performed (Figure 4) with aspiration of 3 mL of clear synovial fluid. There was no evidence of microbial infection nor malignancy from the fluid sample. She was reviewed in clinics after aspiration but her symptoms remained. It was thus decided to proceed with surgical exploration of the antecubital fossa.

**Figure 5:**
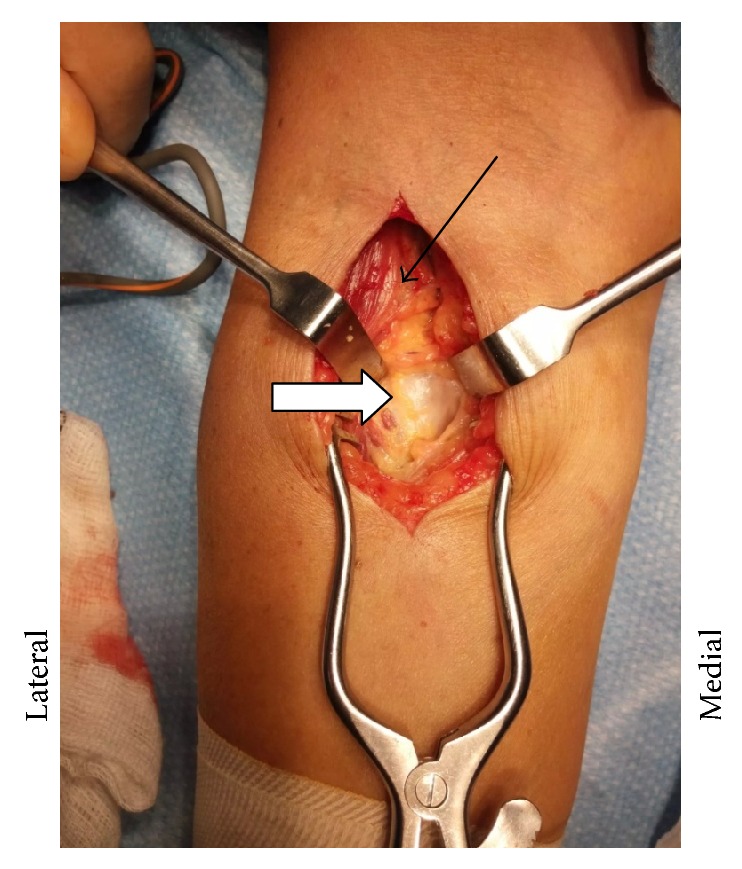
Cubital fossa approach with exposure of bicipitoradial bursa (white arrow). Bicep tendon (thin arrow).

**Figure 6:**
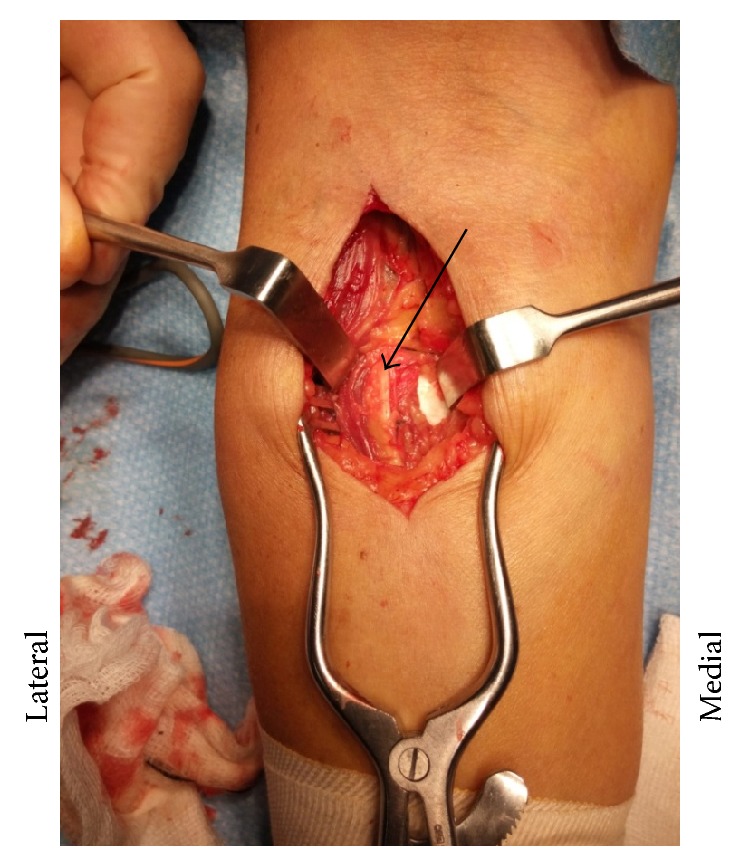
Deeper dissection exposing partial bursa and deep radial nerve (thin arrow).

**Figure 7:**
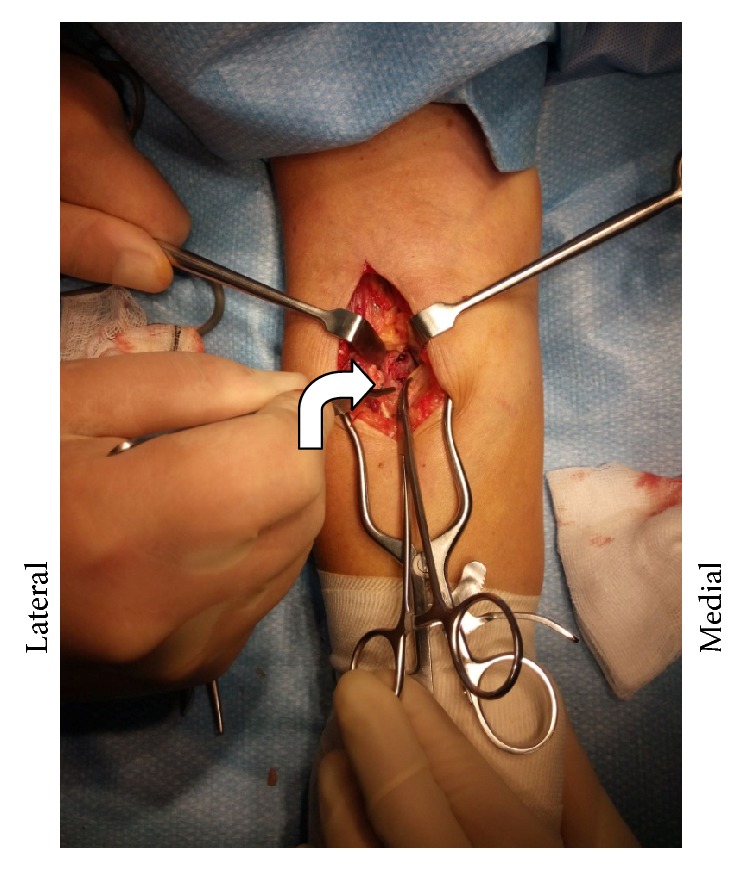
Deep dissection following removal of bursa, exposing radial bone (bent arrow).

Under tourniquet, a linear incision over the right antecubital fossa was performed distal and radial to the biceps brachii tendon. A combination of gentle blunt and sharp dissection through tissue layers was employed towards the direction of the biceps tendon insertion until exposure of the synovial bursa and biceps tendon (Figures 5 and 6). The superficial and deep radial nerve branches were identified and spared throughout the bursa dissection (Figure 6). The surgical margins of the lesion did not extend beyond the radial tuberosity and was engulfing the biceps brachii tendon insertion. Laterally were the supinator and extensor carpi radialis longus muscles; medially was the pronator teres. The synovial cyst and bone mass with a chondral surface were dissected piecemeal and sent for histology. The radius bone was reached after complete removal of the bursa (Figure 7). Soft tissue layers were closed with absorbable sutures (vicryl); skin was closed with nonabsorbable sutures (prolene). Postoperative care involved immobilisation of the elbow to 90 degrees and a shoulder immobilizer. She was discharged the following day with follow-up plan. She was reviewed at outpatient clinics one week after operation and reported improved symptoms. Subsequent histology reported a layer of synovial tissue with a thin overlying cartilage cap confirming an osteochondroma with bicipitoradial bursitis.

**Figure 8:**
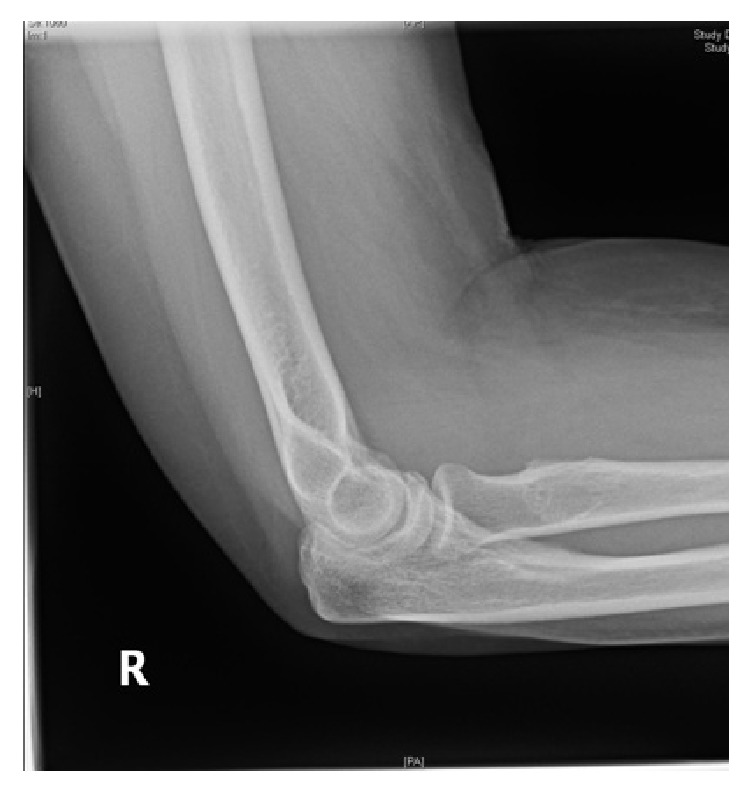
Plain radiograph at followup.

She was followed up after one month and then six months with near complete pain cessation along with improvements in ranges of elbow flexion and pronation. Follow-up plain radiographs show disappearance of the lesion (Figure 8).

## 3. Discussion

Osteochondromas occurring at the elbow are uncommonly reported [7]. Our case was atypical, being a solitary lesion diagnosed at the age of fifty. Her gender was also atypical as a male predominance of 3 : 1 is also reported. Specific to our case report, Orlaw (1891) as cited in Unni [9] coined the term exostosis bursata, which described a thickened bursa formation over the cartilaginous cap of osteochondromas as a rare complication [8]. Radiographic imaging usually demonstrates cortical and medullary bone being in continuity with underlying parent bone [6].

The bicipitoradial bursa is located between the distal biceps tendon and the radial tuberosity. Its function is to allow free movement of biceps tendon during pronation and supination of the forearm. The anatomy of the bicipitoradial bursa was accurately described by Skaf et al. [19]. Histology reveals easy visualization of the posterior wall of the bursa (close to the cortex of the radius), but the anterior wall is hard to distinguish from the paratenon of the biceps tendon. Repetitive mechanical trauma with recurrent pronation and supination can provoke bursitis. Pain with pronation occurs as radial tuberosity rotates posteriorly, compressing the bursa between itself and biceps tendon [19].

Radiology is helpful in furthering the diagnostic process, our case report being a case in point. Sofka and Adler [20] propose that knowledge of the regional anatomy and understanding of the typical sonographic appearance of cubital bursitis are satisfactory for diagnosis and that no additional imaging such as MRI and CT is required. A therapeutic aspiration and injection of steroids into the bursa can be performed at the same time as the diagnostic examination, with pain relief and safe decompression of the bursa, using sonography to guide the needle and avoid regional neurovascular structures [20].

CT scans sometimes detect a rim-enhancing mass adjacent to the radial insertion of biceps tendon. MR imaging findings are a high-signal-intensity material distending bicipitoradial bursa and a relationship between fluid and biceps tendon [21].

For both osteochondromas and bicipitoradial bursitis as separate entities, surgery is a viable option. Liessi et al. [22] demonstrated that surgical resection of bursa is an end stage treatment option after failed conservative management for bursitis. Mirra [23] states that complete resection of osteochondromas should be performed to prevent recurrence; however there is a paucity of evidence in the literature to document the natural evolution of excised osteochondromas apart from Humbert et al. [24] who reported local recurrence to be a rarity. An important point is that there is consensus of no justification for the prophylactic excision of asymptomatic osteochondromas [5].

## 4. Conclusion

Osteochondromas and bicipitoradial bursitis are known causes of antecubital fossa masses and pain. When approaching cubital fossa masses, the initial focus is to exclude a malignancy. Our case report highlighted a diagnostic pathway which ultimately led to surgery following a trial of conservative treatment. To our knowledge there is only one other case report of an osteochondroma at the proximal radius with a secondary bicipital bursitis [7].
